# A synthetic hydrogel for the high-throughput study of cell–ECM interactions

**DOI:** 10.1038/ncomms9129

**Published:** 2015-09-09

**Authors:** Andrew D. Rape, Mikhail Zibinsky, Niren Murthy, Sanjay Kumar

**Affiliations:** 1 Department of Bioengineering, University of California, Berkeley, California 94720, USA

## Abstract

It remains extremely challenging to dissect the cooperative influence of multiple extracellular matrix (ECM) parameters on cell behaviour. This stems in part from a lack of easily deployable strategies for the combinatorial variation of matrix biochemical and biophysical properties. Here we describe a simple, high-throughput platform based on light-modulated hyaluronic acid hydrogels that enables imposition of mutually independent and spatially continuous gradients of ligand density and substrate stiffness. We validate this system by showing that it can support mechanosensitive differentiation of mesenchymal stem cells. We also use it to show that the oncogenic microRNA, miR18a, is nonlinearly regulated by matrix stiffness and fibronectin density in glioma cells. The parallelization of experiments enabled by this platform allows condensation of studies that would normally require hundreds of independent hydrogels to a single substrate. This system is a highly accessible, high-throughput technique to study the combinatorial variation of biophysical and biochemical signals in a single experimental paradigm.

## Abstract (web summary)

Multiple extracellular matrix parameters influence cellular behaviour, but it is difficult to dissect their cooperative contributions. Here the authors describe a hydrogel system in which ligand density and substrate stiffness can be tuned orthogonally to study the contribution of combinations of these parameters simultaneously.

## Graphical abstract

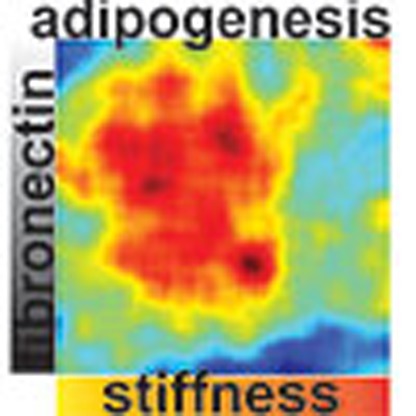

The extracellular matrix (ECM) strongly influences cell and tissue behaviour in disease and development^1^,^2^,^3^,^4^,^5^,^6^,^7^,^8^,^9^. One of the most important functions of the ECM is to provide an adhesive substrate to which integrins and other adhesion receptors bind, which can in turn activate pro-survival signalling cascades in anchorage-dependent cells. In addition to supporting cell adhesion, the physical properties of the ECM serve as an important indirect signal that strongly influences cell behaviour^10^. Because the ECM *in vivo* presents tissue cells with a vast array of interdependent signals, the field’s understanding of this regulation could be accelerated by engineering increasingly complex and high-throughput discovery systems for well controlled, combinatorial investigation of matrix properties. Synthetic hydrogels offer an ideal platform to address this challenge, because they may be specifically engineered with chemical handles to enable spatial patterning of mechanical properties or attachment of proteins that may be used to explore a multitude of matrix conditions in parallel while capturing heterogeneities present within the *in vivo* microenvironment^11^,^12^,^13^,^14^. The use of light as a means to spatially localize chemical reactions in hydrogels is extremely versatile, and has been used in hydrogels to uncage protected reaction sites^13^, induce increased cross-linking^14^ or induce matrix degradation^12^. While these approaches excel at creating gradients and other patterns of single parameters (e.g. stiffness and ligand density), it remains extremely challenging to simultaneously and independently pattern multiple parameters at once. This capability is important because these parameters may interact to influence cell function in complex ways that may not be easily predicted from studies in which parameters are modulated in isolation^9^. While investigators have begun to leverage advanced microarray and microfluidic technologies to create hydrogel systems in which combinations of biochemical signals may be combinatorially varied^15^, there remains a significant unmet need for platforms that can assess combinations of biochemical and biophysical signals that are accessible to a broad swath of cellular researchers. The recent development and application of tunable matrix systems based on heparin^16^,^17^,^18^ and various glycosaminoglycans^19^ suggest that polysaccharides may serve as a valuable framework with which to create such platforms.

In this work, we create a hyaluronic acid (HA) based hydrogel whose stiffness and matrix ligand density can be systematically manipulated with distinct wavelengths of light, which enables us to orthogonally pattern gradients of substrate stiffness and fibronectin density. We use this system to investigate the regulation of cell phenotype as a function of these matrix parameters. Specifically, we find that the oncogenic miR18a is nonlinearly regulated by substrate stiffness and fibronectin density in human glioblastoma (GBM) cells, and that the extent of these non-linearities can be altered by soluble, exogenous factors secreted by other cell types. Finally, we show that the high-throughput nature of this system significantly reduces the number of independent experiments conducted with traditional serially fabricated hydrogels to achieve the same information content.

## Results

### Patterning stiffness and ligand density on HA hydrogels

**Figure 1:**
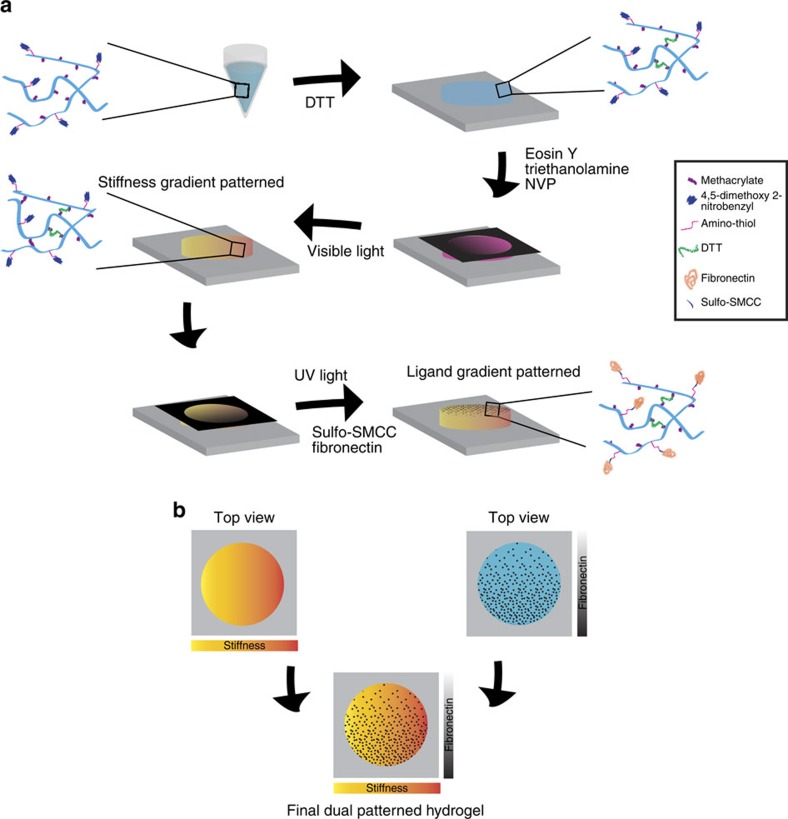
Schematic for design and execution of a high-throughput cell–ECM analysis gel. DMNBAT-HA-methacrylate is crosslinked with DTT to form an insoluble hydrogel attached to a glass coverslip. Next, the gel is incubated in an eosin Y-based photoinitator and exposed to visible light through a gradient photomask creating free-radical based polymerization in the presence of light, resulting in a gradient of elasticity in the gel (**a**). The gel is then extensively washed and exposed to ultraviolet light through a gradient photomask rotated at 90 degrees from the stiffness pattern. The ultraviolet light results in spatially selective exposure of thiol residues that can be conjugated to fibronectin through the heterobifunctional cross-linker sulfo-SMCC (**a**). This process results in orthogonal gradients of both stiffness and fibronectin concentration in a single hydrogel (**b**).

In this report, we sought to create a simple light-controlled hydrogel system in which multiple matrix parameters could be independently varied as means to conduct high-throughput mechanobiology within a single hydrogel. We focused on hydrogel stiffness and matrix ligand density, because both parameters exert strong and sometimes non-linear effects on cell adhesion, motility and lineage commitment^10^. We chose to work with an HA backbone because of its high versatility with respect to chemical functionality and its ubiquity as an implantable biomaterial and tissue engineering scaffold^20^,^21^. We controlled the HA ECM protein ligand density and stiffness independently and in a spatially controlled manner by modifying HA with chemical handles that could be probed by different wavelengths of light. Our reasoning was that continuous patterning of ligand density and stiffness in orthogonal directions would result in a culture system in which a cell’s microenvironmental conditions would be encoded by its position on the gel, such that a significant number of ligand-stiffness combinations could be tested in parallel on a single surface. We stiffness-patterned HA gels based on a two-step Michael addition/light-based crosslinking procedure (Fig. 1)^14^; starting with highly methacrylated HA polymers (50% modification of hydroxyl groups; Supplementary Fig. 1) that were modified for ligand conjugation as described below, we formed a compliant (500 Pa) HA hydrogel surface via dithiothreitol (DTT)-mediated Michael addition reactions^22^, choosing cross-link densities expected to leave ample unreacted methacrylate groups for subsequent functionalization. We then stiffness-patterned these gels by adding the radical initiator eosin Y^23^ and irradiating the gel with visible light through a patterned photomask to initiate additional local cross-linking. This in turn created a gradient of light exposure of the photoinitiator in the gel and selective stiffness-patterning.

We chose to use an uncaging strategy to spatially confine protein binding via a protected chemical handle that is liberated for reactions after exposure to ultraviolet light^13^. The caging strategy was accomplished using a novel photo-conjugation reagent, 4,5-dimethoxy 2-nitrobenzyl-aminothiol (DMNBAT). We chose the 4,5-dimethoxy 2-nitrobenzyl functional group for caging due to its increased stability and higher uncaging efficiency at longer ultraviolet lengths relative to its non-methoxy-modified analogue (Supplementary Fig. 1), and a thiol as our reactive handle due to its specific and fast reactions with maleimide cross-linkers. DMNBAT was synthesized by reacting 2-(Boc-amino)ethanethiol with 4,5-dimethoxy 2-nitrobenzyl bromide, followed by deprotection of the Boc group with HCl (Supplementary Fig. 1b). We then appended DMNBAT to HA-methacrylate backbone before gel cross-linking using carbodiimide chemistry. With this strategy we were able to modify ∼15% of the carboxy groups with DMNBAT, as calculated from relative peak areas in the 1H-NMR spectrum (Supplementary Fig. 1e). Ultraviolet–visible spectral analysis clearly shows nitrobenzyl attachment to the HA backbone, indicated by an absorption peak around 365 nm (Supplementary Fig. 2). We then used the resulting product (DMNBAT-HA-methacrylate) to perform DTT- and eosin Y-mediated cross-linking chemistry to form stiffness-patterned gels as described above.

To spatially pattern ECM ligands, we exposed DMNBAT-HA-methacrylate hydrogels to broad-band ultraviolet light through a gradient photomask, then immediately inundated the gels with dissolved sulfo-sulfosuccinimidyl 4-(*N*-maleimidomethyl)cyclohexane-1-carboxylate (SMCC), a heterobifunctional cross-linker that is terminated by a maleimide group (thiol-specific reactivity) and sulfo-*N*-hydroxysuccinimide group (amine specific reactivity). The maleimide is expected to selectively attach to the exposed thiol only in regions exposed to ultraviolet light. After completion of this maleimide-thiol-based patterning, we conjugated fibronectin to the gels using *N*-hydroxysuccinimide-amine chemistry (Fig. 1).

### Orthogonal patterning of stiffness and ligand density

**Figure 2:**
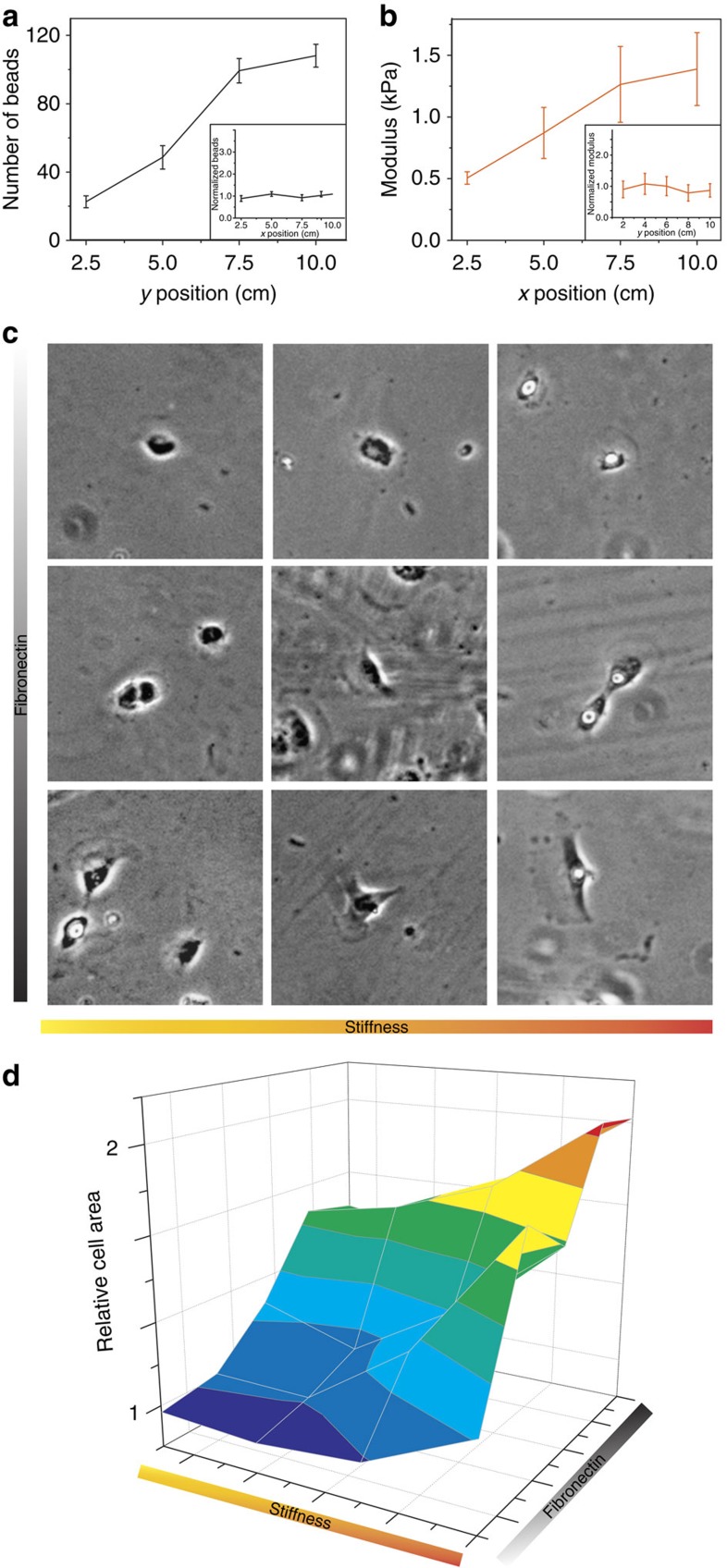
Characterization and validation of orthogonal patterning of stiffness and ligand density. Fibronectin was passively adsorbed to latex beads before conjugation to the gel. Phase contrast microscopy at a magnification of × 20 was used to visualize bead density at different regions in the gel. Beads were automatically counted after manually thresholding the images. Each *y* position (parallel to the gradient) data point consists of the average of 10 different locations along the *x* axis (perpendicular to the gradient) (**a**). To test orthogonality, bead numbers were calculated from four different locations along the *x* axis (perpendicular to the gradient) and normalized to the average value of the respective *y* position (**a**, insert). Elastic moduli were extracted from a modified Hertz model of atomic force microscopy indentation at four positions along the length of gradient (**b**). Each *x* position (parallel to the gradient) data point consists of the average of more than 16 different locations along the *y* axis (perpendicular to the gradient). To test orthogonality, elastic moduli were calculated from five different locations along the *y* axis (perpendicular to the gradient) and normalized to the average value of the respective *x* position (**b**, insert). To test whether these gradients were sufficient to instruct cell behaviour, we plated U373-MG cells on patterned hydrogels and allowed them to adhere (**c**). In soft regions with low fibronectin, cells remained very rounded and rarely exhibited lamellipodia. In contrast, when cells encountered high fibronectin and high stiffness regions of the hydrogel, nearly all cells spread extensively and developed large lamillipodia. A surface plot of cell area was derived from measuring the area of cells at 20 regularized locations on the patterned hydrogel (**d**). At least 10 cells from each location were quantified. Cell size was normalized to the size of the cell on the softest, lowest fibronectin location of the gel. Error bars are s.e.m.

As a test of our ability to independently pattern stiffness and ECM ligand density, we sequentially performed these procedures to produce continuous gradients of stiffness and ligand density oriented in perpendicular directions. To quantitatively assess fibronectin attachment, we treated the gel with fibronectin-coated latex beads following ultraviolet exposure and sulfo-SMCC conjugation and counted the number of bound beads using phase contrast microscopy (Supplementary Fig. 3). These measurements revealed a near-linear increase in bead density across the length of the gradient, with a roughly five-fold difference between the highest and lowest bead densities (Fig. 2a). To verify stiffness-patterning, we used atomic force microscopy to indent the gel in four positions spaced 2.5 mm apart along the stiffness gradient and extracted Young’s moduli by fitting the resulting force-extension curves to a Hertz model^24^. The gel exhibited a nearly linear increase in stiffness across the gradient and the stiffest region of the gel was about three times stiffer than the soft regions (Fig. 2b).

We next evaluated the stiffness and fibronectin density of these gels in the direction perpendicular to the direction of the gradient of interest in dual-patterned gels. There was no significant difference in stiffness at different locations perpendicular to the stiffness gradient (Fig. 2b, insert). When we investigated the orthogonality of fibronectin patterning using bead adhesion, we similarly failed to find any systematic position-dependent variations in the number of beads (Fig. 2a, insert), indicating the presence of the stiffness gradient does not affect the uniformity of the fibronectin gradient. We then confirmed that this conjugation strategy does not compromise presentation of adhesive ligands by successfully detecting immunofluorescence staining against the arginylglycylaspartic acid (RGD) peptide, which has previously been validated as a measure of ligand accessibility (Supplementary Fig. 4)^25^.

### Dual-gradient hydrogels control glioma cell spreading

To test whether the dynamic range of this system is large enough to elicit a cell response, we cultured U373-MG human GBM cells to our dually patterned hydrogels and measured cell spreading, which we have previously shown depends strongly on ECM stiffness and ligand density^26^,^27^. To determine how pairwise combinations of stiffness and fibronectin density regulate cell spreading, we measured the cell spread area at 20 uniformly-spaced locations across the gel. As expected, we observed that cell spread area increases with fibronectin density for all stiffness values. Similarly, increasing substrate stiffness increased spread area at all given fibronectin densities. These two inputs were synergistic, as the largest spread areas were observed when cell adhesion and substrate stiffness were maximized (Fig. 2c,d)^9^.

### Stiffness and ligand density cooperatively regulate miR18a

**Figure 3:**
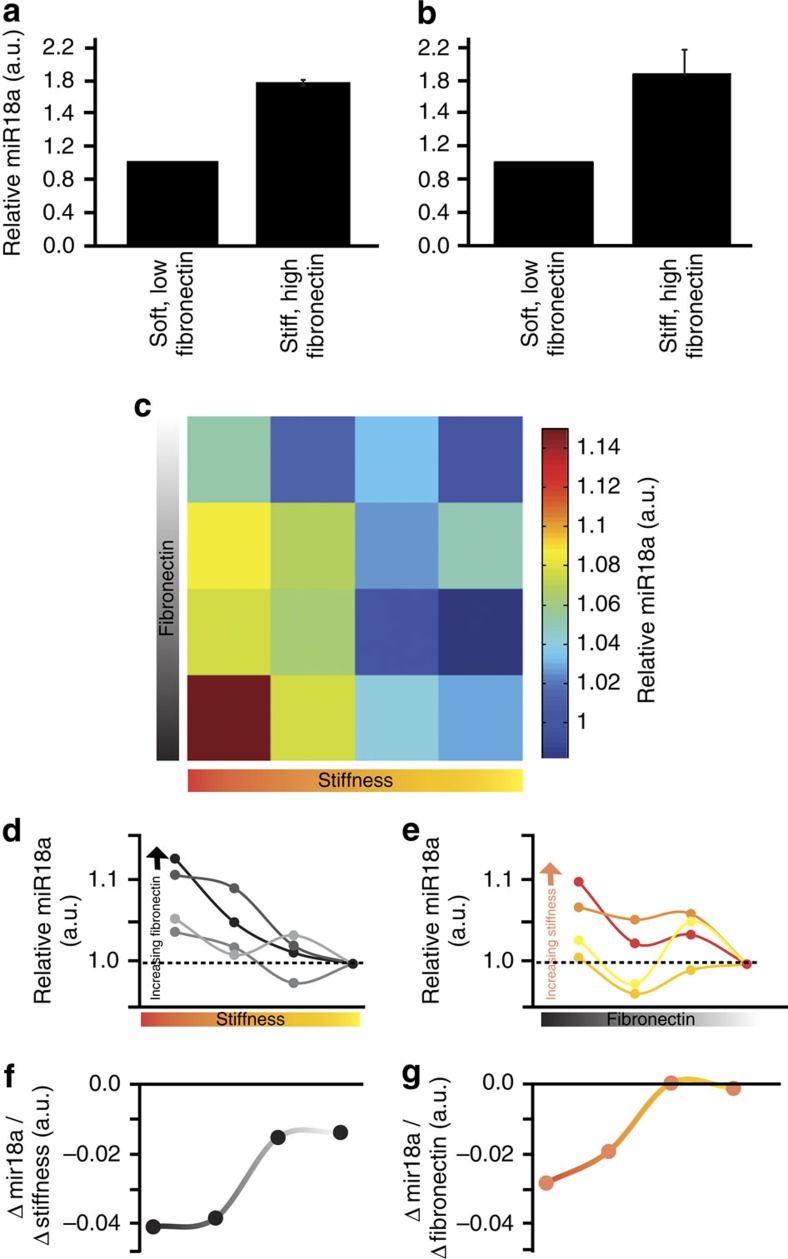
ECM-sensitive regulation of miR18a determined using dual gradient patterned hydrogels. U373-MG cells were incubated with probes for miR18a for 16 h on HA-based hydrogels. MiR18a expression was quantified by taking the sum of the fluorescence of the miR18a probe in an individual cell. Cells cultured on stiff, fibronectin-rich substrates showed elevated miR18a expression relative to soft, fibronectin-deficient surfaces (**a**). These results were confirmed by evaluating the expression of miR18a by reverse transcription–quantitative PCR (RT-qPCR), which was normalized with an internal control (ribosomal 18S RNA; **b**). MiR18a expression was then quantified at 16 matrix stiffness-ligand combinations. (**c**) Iso-fibronectin curves (light red to dark red equals low fibronectin to high fibronectin) show that substrate stiffness regulates miR18a expression at all fibronectin densities tested (**d**). Similarly, iso-stiffness curves (light blue to dark blue equals low stiffness to high stiffness) show that fibronectin density only regulates miR18a expression at high stiffness (**e**). Each curve in *d* and *e* is normalized to the softest or lowest fibronectin density for each iso-curve. The gradient of each iso-curve further demonstrates that substrate stiffness regulates miR18a expression at all fibronectin densities (**f**; uniformly negative gradient values), and that fibronectin density only regulates miR18a expression on stiff substrates (**g**). *N*>85 cells for each stiffness-ligand combination measurement of miR18a. Errors bars are s.e.m. for fluorescence-based miR18a measurements (**a**) and represent the variability in the ΔΔCt method for the RT-qPCR analysis (**b**).

MicroRNAs are small nucleic acid sequences that have profound effects on gene transcription and protein translation^28^. One specific microRNA, miR18a, is both increasingly recognized as highly oncogenic and is expressed aberrantly in human GBM tumours^29^. Interestingly, recent work demonstrates that miR18a expression is increased when matrix stiffness increases in both *in vitro* and *in vivo* paradigms of breast cancer^30^. To demonstrate the power of our high-throughput patterning technology, we focused on miR18a as a model target with which to investigate combinatorial regulation of microRNA levels by substrate stiffness and ECM-ligand density. We measured miR18a expression using a fluorescence-based assay for microRNA expression. Briefly, live cells are incubated with a fluorescent probe, which then binds to the microRNA of interest and causes an increase in fluorescence of the probe due to the liberation of a previously quenched fluorophore. To determine whether miR18a is indeed regulated by ECM parameters, we cultured U373-MG cells on our modified-HA hydrogels that were uniformly patterned with the two most extreme conditions: either stiff and fibronectin-rich or soft and fibronectin-deficient. Using the fluorescence-based assay, we found an approximately twofold increase in miR18a expression on stiff, fibronectin-rich matrices (Fig. 3a). We then confirmed this result with reverse transcription–quantitative PCR, where we found a very similar twofold increase under the same conditions (Fig. 3b).

After establishing the novel finding that miR18a is regulated by ECM parameters in a glioma cell line, we next wanted to quantitatively understand the regulation of miR18a expression as a function of fibronectin density and substrate stiffness. We cultured U373-MG cells on dual–patterned gradient gels, imaged cells that were incubated in a miR18a fluorescent reporter at 16 evenly spaced locations on the gel surface, and quantified the expression at each location (Fig. 3c). U373-MG cells showed significant variation in miR18a expression as a function of both fibronectin density and substrate stiffness. For each fibronectin concentration, we plotted miR18a expression as a function of substrate stiffness. Each iso-fibronectin curve was independently normalized to the softest condition in that family (Fig. 3d). At every fibronectin concentration, we observed an increase in miR18a expression as a function of substrate stiffness. We performed similar analysis at each iso-stiffness (Fig. 3e), and observed substantial regulation of miR18a expression only at the two highest stiffnesses. We next plotted the gradient of the best-fit line for each iso-fibronectin curve (Fig. 3f) and each iso-stiffness curve (Fig. 3g). For all fibronectin densities, the slope is negative, indicating that substrate stiffness exerts control on miR18a expression for all fibronectin densities encompassed in our gel. In contrast, on the two softest regions of the gel, the derivative of the best-fit lines of the iso-stiffness curves are essentially zero, indicating that fibronectin concentration does not regulate miR18a expression on soft substrates. We then plotted the correlation between independently measured miR18a expression and cell spreading area and found positive correlations between the two variables on all but the softest substrates (Supplementary Fig. 5). Together, these analyses reveal that miR18a is maximally mechanosensitive under specific matrix conditions and that the ECM regulates miR18a regulation in complex and nonlinear ways.

The finding that matrix parameters regulate a pro-oncogenic miRNA in such a complex, nonlinear fashion made us wonder whether such parameters might also modulate responses to soluble paracrine signals found within the tumor microenvironment (and, reciprocally, whether these microenvironmental cues might influence matrix sensing). Tumor associated macrophages strongly influence the size and progression of human GBM tumours, making macrophage-derived signals a natural candidate to investigate. We therefore cultured U373-MG cells on dual-patterned hydrogels in both standard growth medium and medium that had been conditioned by cultured C8-B4 macrophages. When we revisited our cell spreading measurements, we found that U373-MG cells display markedly decreased sensitivity to substrate stiffness at all concentrations of fibronectin density, as observed by the similarity in spreading area versus stiffness over the range for a given fibronectin concentration (Supplementary Fig. 6). Most notably, we found that exposure to macrophage-conditioned medium was capable of partially rescuing spreading on soft, high-fibronectin matrices. To confirm this unexpected result, we created soft, uniform (non-patterned) hydrogels with high-fibronectin concentrations and cultured cells in either standard growth medium or macrophage-conditioned medium. Cells exposed to macrophage-conditioned medium indeed exhibited increased spreading relative to cells cultured in normal growth medium (Supplementary Fig. 7). When compared with the strong mechanosensitivity observed for U373-MG cells cultured in standard growth medium, this would imply that macrophages secrete paracrine factors that regulate stiffness-sensing.

**Figure 4:**
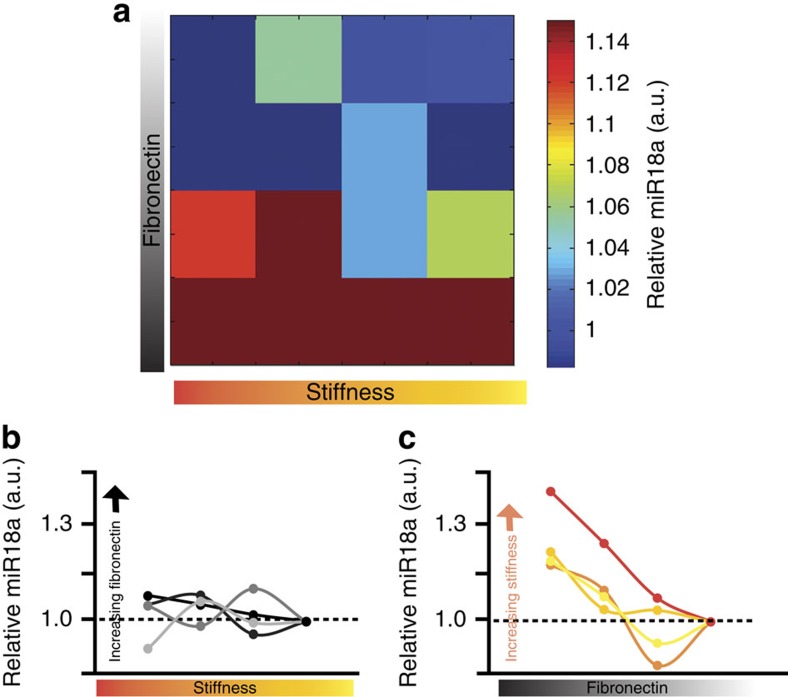
The effect of exogenous, macrophage-derived soluble factors on ECM-sensitive regulation of miR18a. U373-MG cells were incubated with fluorescent probes for miR18a after prior incubation with medium conditioned for 4 days by C8-B4 macrophages. Cells cultured on high-fibronectin locations on the gel showed high miR18a expression, while cells were relatively insensitive to substrate stiffness at all fibronectin densitites. (**a**) Iso-fibronectin curves showed no mechanosensitivity of miR18a expression in cells cultured in macrophage-conditioned medium (**b**). Contrary to the reduction of mechanosensitivity, fibronectin sensitivity remained at all iso-stiffness points when cells were cultured in macrophage-conditioned medium (**c**). *N*>30 cells for each stiffness-ligand density combination measurement of miR18a.

To investigate transcriptional consequences of this reduced stiffness-sensing, we next measured the sensitivity of miR18a expression to substrate stiffness and fibronectin density for U373-MG cells under the influence of macrophage-conditioned medium. Consistent with our cell spreading results, we found that at high-fibronectin concentration, there was qualitatively no change in the expression of miR18a at different stiffnesses, with only modest stiffness sensitivity at low fibronectin densities (Fig. 4a). As with the spreading measurements, miR18a expression in cells exposed to macrophage-conditioned medium was sensitive to fibronectin density at all stiffness. This suppression of stiffness-sensitive miR18a expression is more quantitatively apparent in plots of the expression of miR18a as a function of substrate stiffness at each iso-fibronectin point (Fig. 4b). Conversely, analogous iso-stiffness curves for various fibronectin densities reveal that exposure to macrophage-conditioned medium preserves the sensitivity of mRr18a expression to fibronectin density (Fig. 4c).

### Patterning of mesenchymal stem cell (MSC) differentiation

**Figure 5:**
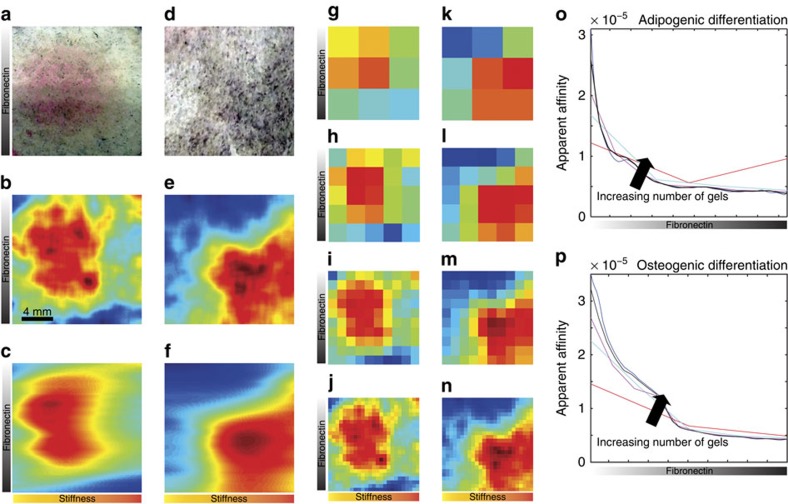
Adipose-derived mesenchymal stem cell differentiation of dual gradient patterned DMNBAT-HA-methacrylate hydrogels. Adipose-derived mesenchymal stem cells were cultured in mixed differentiation medium for 7 days on dual gradient gels. Cells were fixed and stained for liposomes with Oil Red O (**a**) or alkaline phosphatase activity with BCIP/NBT (**d**). Adipogenic differentiation was observed by a red stain in (**a**) on soft regions of the hydrogel whose location is relatively independent of fibronectin concentration. Osteogenic differentiation was observed by a dark purple stain in (**d**) that was strongest in regions of high stiffness and high fibronectin. Individual differentiation images were then averaged together to create composite maps of differentiation ‘hotspots’ (**b**,**e**). The data were reconstructed using a statistical dynamics model that was fitted to the experimental data (**c**,**f**). The experimental data for either adipogenic differentiation (**g**–**j**) or osteogenic differentiation (**k**–**n**) was grouped into bins of varying resolution, from 9 to 625 bins. The importance of high-resolution measurements is demonstrated by the increasingly exponential behaviour of the fitting parameter, apparent affinity, as the number of bins increases (**o**,**p**). Scale bar, 4 mm.

To demonstrate the versatility with respect to different cell types of our material platform, we used this system to study the combinatorial effects of ECM biophysical and biochemical properties on MSC differentiation^31^. We hypothesized that we could use this system to discover the optimal combination of conditions to promote MSC differentiation to a desired lineage. To explore this possibility, we plated adipose-derived MSCs on sequentially gradient-patterned DMNBAT-HA-methacrylate hydrogels. After 1 day in growth medium, we converted to a 1:1 mixture of adipocytic and osteogenic differentiation medium^14^,^32^. After 7 days of exposure to this mixed differentiation medium, cells were fixed and differentiation was assessed with either Oil Red O staining to visualize adipocytic differentiation or 5-bromo-4-chloro-3-indolyl phosphate (NBT/BCIP) to visualize osteogenic differentiation^32^. Osteogenic differentiation was observed when fibronectin density and substrate stiffness were high (Fig. 5b; purple). Conversely, adipogenic differentiation was relatively insensitive to fibronectin density, instead depending much more strongly on substrate stiffness (Fig. 5a; red).

### Information content of dual-patterned hydrogels

To assess the importance of high-throughput measurements in obtaining accurate and complete results, we fit our experimental data to a previously established model that relates the probability of stem cell differentiation as a function of substrate stiffness at a constant ligand density to the affinity of a mechanosensitive differentiation factor to focal adhesions^31^. At each ligand density, we fit our experimental data to the model and observed good agreement between the two (Fig. 5c–f). We then assigned the experimental differentiation data to bins of varying size, ranging from very coarse (9 bins) to fine (625 bins), representing the number of independent, conventionally fabricated gels one would need to make to obtain similar results (Fig. 5g–n). We then fit our experimental data to the differentiation model at different resolution values to determine whether changing the resolution could change the computed fit-parameters. Indeed, at low resolution, the apparent binding affinity parameter, K, decreased only modestly as a function of ligand density for both adipogenic and osteogenic differentiation (Fig. 5o,p; red line). As the resolution of the data increased, we observed that in both differentiation cases, the apparent affinity decreased exponentially as a function of ligand density (Fig. 5o,p; black lines). Thus, we conclude that probing MSC differentiation at high resolution provides quantitative insight into how ECM stiffness regulates the binding affinity of mechanosensors to focal adhesions that might be overlooked in lower-resolution studies featuring serially fabricated hydrogels.

## Discussion

We have developed a hydrogel ECM system in which biochemical and biophysical properties may be varied independently and simultaneously, and we have applied this materials system to the high-throughput study of cell–ECM interactions. This system leverages highly modifiable hydrogels with light-accessible chemical handles to create a platform that systematically explores a two-dimensional space defined by stiffness and ligand density. By using materials whose mechanical properties may be modulated within a physiologic range, our platform builds on past high-throughput platforms for investigating cell–ECM interactions, which have conventionally emphasized robotic deposition of ECM proteins on rigid glass or plastic substrates^33^,^34^,^35^. Moreover, because this approach does not require sophisticated instrumentation or complex conjugation chemistry, it offers an alternative to recent and very elegant approaches based on robotic spotting of reactive poly(ethylene glycol) hydrogels that enable control of ligand density, ligand type and matrix mechanics, but not in high-throughput combinations of ligand and mechanics^36^. The ability to photo-pattern geometrically complex gradients in stiffness and ligand density may also offer new opportunities to study and manipulate tissue development, assembly and morphogenesis *in vivo*.

As a proof of principle, we used this technology to study the synergistic regulation by substrate stiffness and fibronectin density of miR18a expression in U373-MG GBM cells and lineage commitment in adipose-derived MSCs. We found that miR18a expression is nonlinearly regulated by both fibronectin and substrate stiffness, and that the nature of this non-linear regulation can be altered by exogenous factors derived from other cells. This suggests a level of interconnectedness between multiple ECM parameters that invites further study and highlights the importance of conducting measurements in highly multiplexed conditions. Our findings also suggest nonlinear regulation by stiffness and fibronectin density in MSC differentiation. Corroborating previous studies, we find that adipogenic differentiation is favored on soft, fibronectin-deficient matrix conditions, whereas osteogenic differentiation is favoured on stiff, fibronectin-dense conditions^31^,^37^. Because of the highly parallelized nature of our system, we were able to identify and define much more precise matrix conditions that promote each differentiation outcome, which allowed us to observe that adipogenic differentiation is relatively insensitive to fibronectin density, instead depending much more strongly on substrate stiffness over the range of conditions in this experiment.

In conclusion, we have developed a single hydrogel-based platform for the high-throughput study of the synergistic effects of multiple matrix parameters on cell phenotype. Because this technology requires relatively little infrastructure and can be integrated easily with standard and/or advanced molecular and cell biological assays, it may be readily adopted by non-specialists and should facilitate a broader and deeper understanding of how ECM parameters regulate cell behaviour.

## Methods

### Synthesis of DMNBAT

2-(Boc-amino)ethanethiol (1 g, 0.0056, mol) was dissolved in 20 ml of tetrahydrofuran (THF) in a round bottom flask, and 1.1 eqivalents of triethylamine (0.62 g, 0.86 ml) was added to the THF solution. 4,5-dimethoxy 2 nitrobenzyl bromide was dissolved in 20 ml of THF, and was added portionwise to the thiol solution over a period of 1 min. The reaction was covered with aluminium foil and was allowed to stir at room temperature overnight. The THF was rotavapped off, and the resulting residue was dissolved in dichloromethane (DCM) and purified by silica gel column chromatography. Two columns were needed to isolate a pure product: the first used DCM as the eluent, and then a second column was run using ethyl acetate/hexane as the eluent.

The amine-protected DMNBAT was dissolved in dioxane-HCl and stirred for 30 min, the dioxane was rotavapped off and H-NMR analysis was perfomed to assess purity. The recovered solid was then dissolved in a mixture of 90 ml water/10 ml 0.1 N HCl and refluxed for 5 min. The solution was cooled down and extracted two times with 10 ml of DCM, the DCM was dried and vacuum dried after rotavapping. Yield 800 mg, 52% for both steps. 1H NMR (400 MHz): δ, 2.699–2.736 (t, 2H), 2.95 (s, 2H), 3.844 (s, 3H), 3.939 (s, 3H), 4.091 (s, 2H), 7.302 (s, 1H), 7.561 (s, 1H) and 8.261 (s, 3H). 13C NMR: δ, 27.853, 32.790, 38.572, 55.961, 56.566, 108.755, 114.247, 129.122, 139.619, 147.434 and 152.683. Mass spectrometry was performed on an AB SCIEX 3200 QTRAP LC/MS/MS system (AB SCIEX, USA) equipped with a liquid chromatograph system. MS data were analysed using the PeakView software (AB SCIEX, USA). MS (ESI) calculated for C_11_O_4_N_2_SH_16_ ([M+H]^+^) 273.32, found 273.24.

### Synthesis of HA-methacrylate

HA (MW 66–90 kDa; Lifecore Technologies) was dissolved at a concentration of 1 wt% in deionized water. The solution was placed in an ice bath and a sixfold molar excess of methacrylic anhydride (Sigma; relative to the HA disaccharide repeat unit) was added dropwise to the solution^22^. The pH was rendered >8 with concentrated NaOH. The pH was held >8 as the reaction continued overnight at 4 °C. The next day, twofold molar excess of methacrylic anhydride was added dropwise to the solution, which was then maintained at 4 °C for 2 more days. At the conclusion of the reaction, the remaining methacrylic anhydride was allowed to phase separate from the aqueous HA and was removed. HA was precipitated from the aqueous solution by adding fourfold volumetric excess of cold acetone to the HA-methacrylate. After centrifugation at 1,000*g* for 10 min, the acetone was removed and the remaining HA-methacrylate was air dried in a fume hood. HA-methacrylate was then dissolved at an approximate concentration of 45 mg ml^−1^, flash frozen and lyophilized.

### Synthesis of DMNBAT-HA-methacrylate

HA-methacrylate was dissolved in deionized water at a concentration of 5 wt%. At room temperature, an equimolar amount of DMNBAT, a twofold molar excess of *N*-(3-dimethylaminopropyl)-N′-ethylcarbodiimide hydrochloride (EDC; Sigma), and 0.002 fold of triethylamine (Sigma) were added (all molar amounts were relative to HA disaccharide repeat unit). The reaction was allowed to proceed overnight. At the conclusion of the reaction, the reacting solution was dialyzed for 4 days in distilled water with daily water changes. The remaining solution was then flash frozen and lyophilized.

### Chemical characterization of DMNBAT-HA-methacrylate

The degree of functionalization of DMNBAT-HA-methacrylate was obtained by dissolving it at 5 mg ml^−1^ in D_2_O. NMR spectra were collected at 400 MHz using a Bruker Avance AVB-400 instrument. The degree of methacrylation was then calculated as the ratio of the vinylic protons from the methacrylate group to the *N*-acetyl methyl protons from the HA backbone, normalized to the number of protons per group (Supplementary Fig. 1d)^22^. The degree of DMNBAT functionalization was calculated as the ratio of the aromatic protons from the nitrobenzyl group to the *N*-acetyl methyl protons from the HA backbone, normalized to the number of protons per group (Supplementary Fig. 1e)^13^.

### Preparation of two-dimensional hydrogels from DMNBAT-HA-methacrylate

HA hydrogels were prepared on flat glass coverslips which were rendered hydrophobic by treatment with hydrophobic solution (OMS Optichemicals), which aided in adhering the gel to glass slide. DMNBAT-HA-methacrylate was mixed at a 1:1 ratio with HA-methacrylate and dissolved in PBS at 5 wt%. DTT (Sigma) was added at a ratio of 0.3 thiol units relative to the HA-repeat units. The solution was pipetted on to the hydrophobic glass coverslip and covered with a hydrophilic glass coverslip, which had been previously exposed to plasma, to form a gel sandwich. The HA was polymerized 12 h at 37°C inλ a humidified environment, after which the top coverslip was carefully removed to reveal a flat hydrogel of nominal thickness 100 μm. The solution was soaked in PBS for 5 h to remove unreacted DTT.

### Design and fabrication of photomask

Gradient patterns were created in Photoshop (Adobe) linearly ranging from 90 to 10% black. Gradient masks were printed with a LaserJet Pro 400 M401 printer (HP) at a resolution of 1,200 d.p.i. on transparencies.

### Gradient fibronectin density patterning of DMNBAT-HA-methacrylate hydrogels

A photoinitiator solution was made that consisted of 9.7 μg ml^−1^ eosin Y (Sigma), 0.97% triethanolamine (Sigma), 0.97% *N*-Vinylpyrrolidone (NVP; Sigma) in PBS. Photoiniator (500 μl) was added to a carefully dried DMNBAT-HA-methacrylate hydrogel and incubated for 30 min at room temperature. The excess photoinitiator was then carefully removed with a paper towel and a gradient transparency photomask was brought into manual contact with the gel. The gel was exposed to visible light from a MagLite flashlight for 25 min, after which the photomask was carefully removed and the hydrogel was immediately washed three times in PBS to remove remaining photoinitiator.

### Gradient stiffness pattering of DMNBAT-HA-methacrylate hydrogels

DMNBAT-HA-methacrylate hydrogels were carefully dried with a paper towel and brought into manual contact with a gradient photomask. The gel was exposed to broad ultaviolet light with a Sunray 400 SM UV curing system (UviTron) for 5 min. The gel was then immediately immersed in 1 mg ml^−1^ sulfo-SMCC (Thermo-Fisher) in PBS. After 30 min, the sulfo-SMCC solution was carefully removed with a paper towel and 0.1 mg ml^−1^ human plasma fibronectin (Millipore) was added to gel and allowed to react for 1.5 h.

### Local mechanical quantification of patterned hydrogels

Dual gradient patterned DMNBAT-HA-methacrylate hydrogels were assessed on an MFP-3D atomic force microscope (Asylum Research). The gels were indented with a pyramid-tipped probe (OTR4; Bruker AFM Probes) with cantilever spring constants of 0.07–0.21 N m^−1^, as measured by thermal calibration. Elastic moduli of the gels were calculated from force curves using a modified Hertz model^24^.

### Cell culture

U373-MG human GBM cells (ATCC HTB-17, which have been reported to share common origins with U251-MG GBM cells) were cultured in DMEM (Invitrogen) with 10% Calf serum advantage (JR Scientific, Inc), 1% penicillin-streptomycin, 1% MEM non-essential amino acids and 1% sodium pyruvate (Sigma)^26^. Human primary adipose derived MSCs were purchased from ATCC and maintained and grown in accordance with their recommendations. Cells were cultured and grown in MSC growth media supplemented with MSC growth supplements (ATCC) and used at passage <5. C8-B4 cells were obtained from ATCC and cultured in DMEM with 10% calf serum, 1% penicillin-streptomycin, 1% MEM non-essential amino acids and 1% sodium pyruvate. Macrophage-conditioned medium was collected after 4 days of culture. For experiments with macrophage-conditioned medium, U373-MG cells were initially seeded to patterned hydrogels in their normal growth medium and allowed to adhere to the gel overnight. The medium was then replaced with macrophage-conditioned medium and measurements were taken 2 days later.

### Differentiation and staining of adipose-derived human MSCs

Adipose-human MSCs were seeded on dual-patterned DMNBAT-HA-methacrylate hydrogels and allowed to grow for 1 day, at which times cells were treated with a mixed differentiation media, consisting of a 1:1 mixture of osteogenic and adipogenic differentiation medias (both StemPro, Life Technologies). Cells were maintained for 7 days in these conditions with media changes every 3 days.

For osteogenic staining, cells were fixed with 4% paraformaldehyde (Sigma) for 10 min at room temperature, permeabilized with 0.2% Triton X-100 (Sigma), then washed extensively with PBS. Osteogenic differentiation was assessed by alkaline phostphatase activity via the SigmaFast BCIP/NBT assay (Sigma). A working solution of BCIP/NBT was prepared as per manufacturer’s instructions, added to the cells, incubated for 10 min, and then washed in PBS extensively. Alkaline phosphatase staining was identified as a dark blue colour.

For adipogenic staining, cells were fixed with 4% paraformaldehyde for 10 min at room temperature, then washed extensively with PBS. Adipogenic differentiation was assessed by Oil Red O staining (Sigma), which stains for lipds in adipocytes. A stock solution of 3 mg ml^−1^ Oil Red O in isopropanol was prepared. Oil Red O stock solution was then mixed at a 3:2 ratio with distilled water and incubated for 10 min at room temperature. After fixation, cells were immersed in 60% isopropanol for 5 min. The isopropanol was then removed and the working solution of Oil Red O was added to the cells and incubated for 10 min at room temperature. The cells were then washed extensively with PBS until there was no red colour remaining in the discarded PBS. Adipogenic differentiation was identified as a red colour.

The images were converted to grayscale and averaged for each differentiation condition. To remove small noise from the data, a Gaussian filter was applied to each averaged image.

### Quantification of fibronectin attachment density

Human plasma fibronectin (0.1 mg ml^−1^) was added to 10% 3-μm mean diameter latex beads (Sigma) and incubated at room temperature for 30 min. The beads/fibronectin was then added to the gel following the sulfo-SMCC reaction, as described above. After extensive washing, phase contrast images of the beads were obtained with a × 20 objective on a phase contrast microscope. Images were then thresholded and counted with ImageJ.

### Fluorescence-based miR18a measurement

U373-MG cells that were previously transduced with cytoplasmic mCherry were incubated with Millipore SmartFlare probes for miR18a at the manufacturer’s recommended concentration for 16 h. Images of the cells at regularized locations on the gel were acquired in the mCherry and miR18a channels with a Prairie Technologies Swept-Field Confocal microscope. Individual cell areas were determined with an automated segmentation algorithm in MATLAB that took as input the mCherry signal. The total miR18a signal in each identified cell was then calculated and normalized to the total mCherry signal.

### Reverse transcription–quantitative PCR

U373-MG cells were grown on dual gradient patterned hydrogels overnight, after which total mRNA was isolated from the cells with an Ambion Cells-to-Ct kit, as per the manufacturer’s instructions. Reverse transcription was performed with primers for miR18a (Applied Biosystems) or universal amplification (iScript; BioRad), with three thermal stages on a BioRad C1000 Thermal Cycler 1(6°C for 30 min, 42°C for 30 min, 85°C for 5 min). Quantitative PCR was performed on either the target or control sample with a BioRad iQ5 quantitative PCR machine with primers for either miR18a (Applied Biosystems) or for 18S ribosomal RNA (control; Applied Biosystems). The Ct of each sample was computed with BioRad software, after which differences between soft and stiff samples were calculated using the ΔΔCt method.

### Microscopy and image analysis

Phase contrast images were acquired on either a Nikon TE200E2 or a Nikon Eclipse Ti microscope equipped with × 10 and × 20 lens. Cells on DMNBAT-HA-methacrylate hydrogels were fixed with 4% paraformaldehyde and imaged with a phase contrast microscope at a magnification of × 10. A 5 × 4 regularized grid that spanned the gel was acquired. Individual cell size for each image was manually drawn and computed with ImageJ.

### Adipose-derived stem cell differentiation modelling

Our model of stem cell differentiation as a function of stiffness was based on a previously described model^31^. Briefly, we assume two possible states of a key differentiation factor *‘X’* which binds with apparent affinity, *K*, at focal adhesions. The free energy of the model depends on the state of *X*, the global prestress (*σ*), and the volume of the cell (*V*). We assume that the cell has constant volume at all conditions. The probability of lineage commitment as a function of elasticity of the substrate at a constant ligand concentration can then be written as:




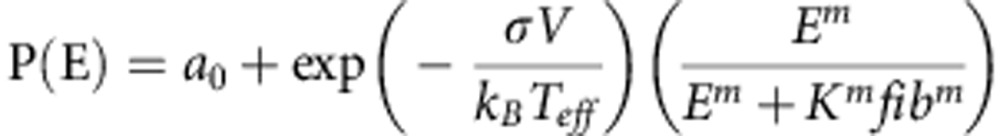




where *fib* is the ligand concentration, *K*_B_ is the Boltzman constant. We used *T*_eff_, *m* and *K* as free fitting parameters to optimize the fit of the model to our experimental at each ligand concentration.

### Statistics

All images and data are representative of the results of at least two or more independent biological experiments. Statistical significance was determined using one-way analysis of variance followed by the Tukey–Kramer HSD test for multiple comparisons. The significance level was set at *P*=0.05.

## Additional information

**How to cite this article:** Rape, A. D. *et al.* A synthetic hydrogel for the high-throughput study of cell–ECM interactions. *Nat. Commun.* 6:8129 doi: 10.1038/ncomms9129 (2015).

## Supplementary Material

Supplementary Information Supplementary Figures 1-7
