# Supplementary material for: A synthetic hydrogel for the high-throughput study of cell–ECM interactions
Source: Nat Commun. 2015 Sep 9;6:8129. doi: 10.1038/ncomms9129 (PMC4566157; doi:10.1038/ncomms9129)
Supplement: Supplementary Information — Supplementary Figures 1-7 [file ncomms9129-s1.pdf]

a

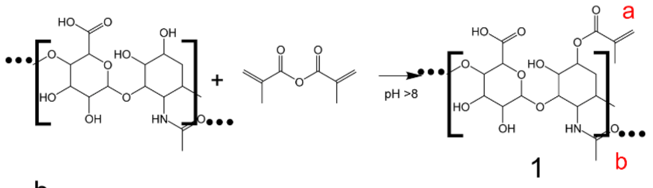

b

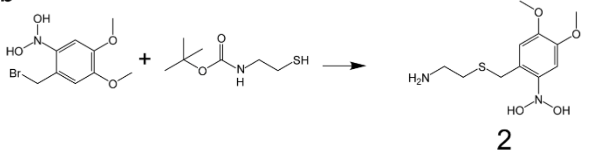

c

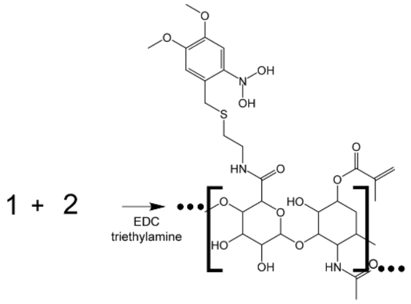

d

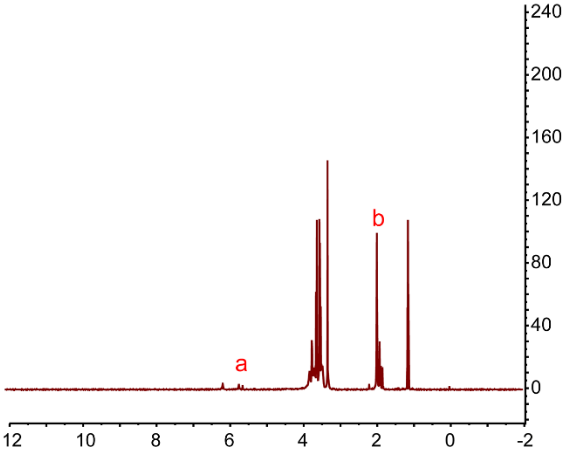

e

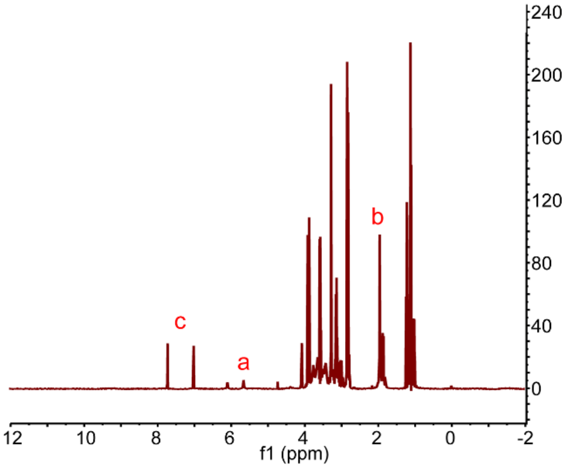

Supplementary Figure 1. Synthesis of DMNBAT-HA-methacrylate. High molecular weight HA was reacted with methacrylic anhydride at elevated pH resulting in the conversion of hydroxyl groups on the HA backbone to methacrylate groups (a, **product 2;** d). 2-(Boc-amino)ethanethiol was mixed in molar equivalency with 4,5-dimethoxy 2-nitrobenzyl bromide and reacted overnight at room temperature. Removal of the Boc protecting group resulted in a nitrobenzyl protected aminothiol (b, **product 2**). **Product 1** was conjugated to **product 2** via carboxy groups on the HA backbone and the amine group on **product 2** with EDC-based chemistry to yield the final DMNBAT-HA-methacrylate (c). 1H-NMR of product 1 shows the presence of methacrylic modifications of the HA backbone (d) 1H-NMR of DMNBAT-HA-methacrylate revealed the presence of the aromatic protons from the nitrobenzyl group between 7-8, vinylic protons from the methacrylate group between 5.5-6, and control N-acetyl methyl protons from the HA backbone (e).

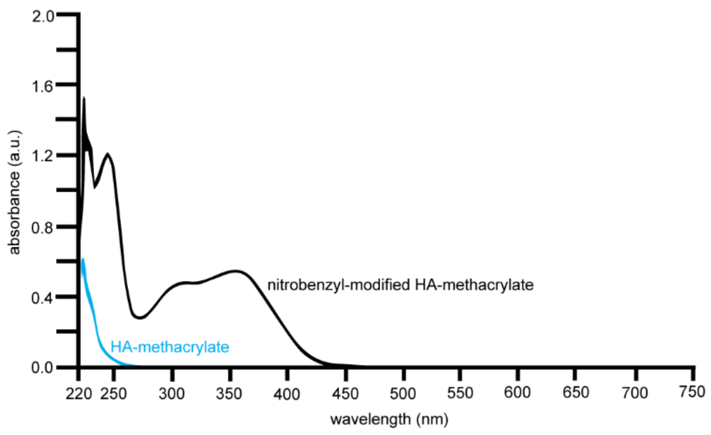

Supplementary Figure 2. UV-vis spectrums of methacrylated HA and nitrobenzyl-methacrylated HA. UV-vis spectra were collected for both the intermediate HA-methacrylate and final product nitrobenzyl-modified HA-methacrylate. Nitrobenzyl-modified HA-methacrylate showed a strong absorption at ~360 nm, corresponding to the expected absorption for nitrobenzyl moieties.

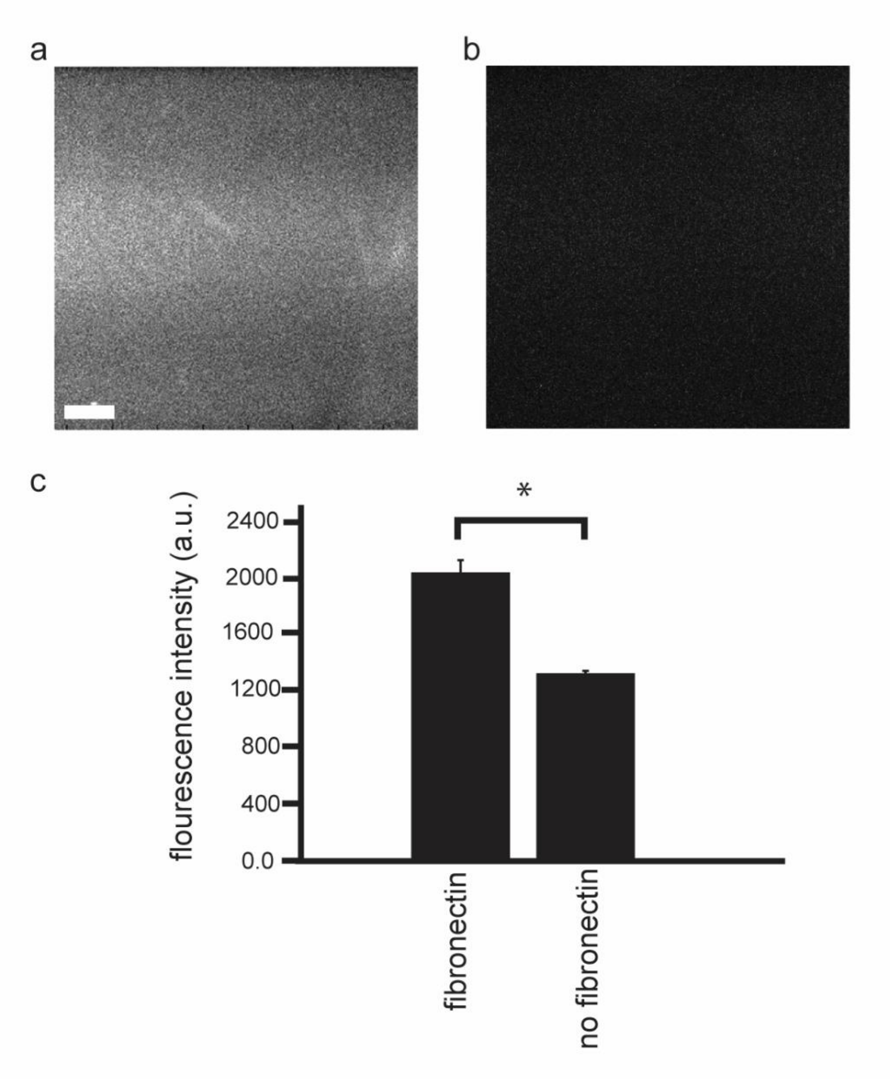

Supplementary Figure 3. Accessibility of cell-adhesive domains on fibronectin conjugated hyaluronic acid hydrogels. DMNBAT modified were conjugated fibronectin and RGD accessibility on fibronectin was accessed by immunofluorescent staining against the RGD peptide sequence. Signficant fluorescence was observed on fibronectin conjugated hydrogels (a), while very little fluorescence was observed on control gels that were exposed to light but the cross-linker sulfo-SMCC was omitted from the process (b). Quantification of average fluorescent intensity reveals a nearly two-fold increase in fluorescent intensity over control gels, indicating that the cell adhesive RGD peptide domain is accessible on fibronectin. *=$p<0.001$. Scale bar, 50 microns.

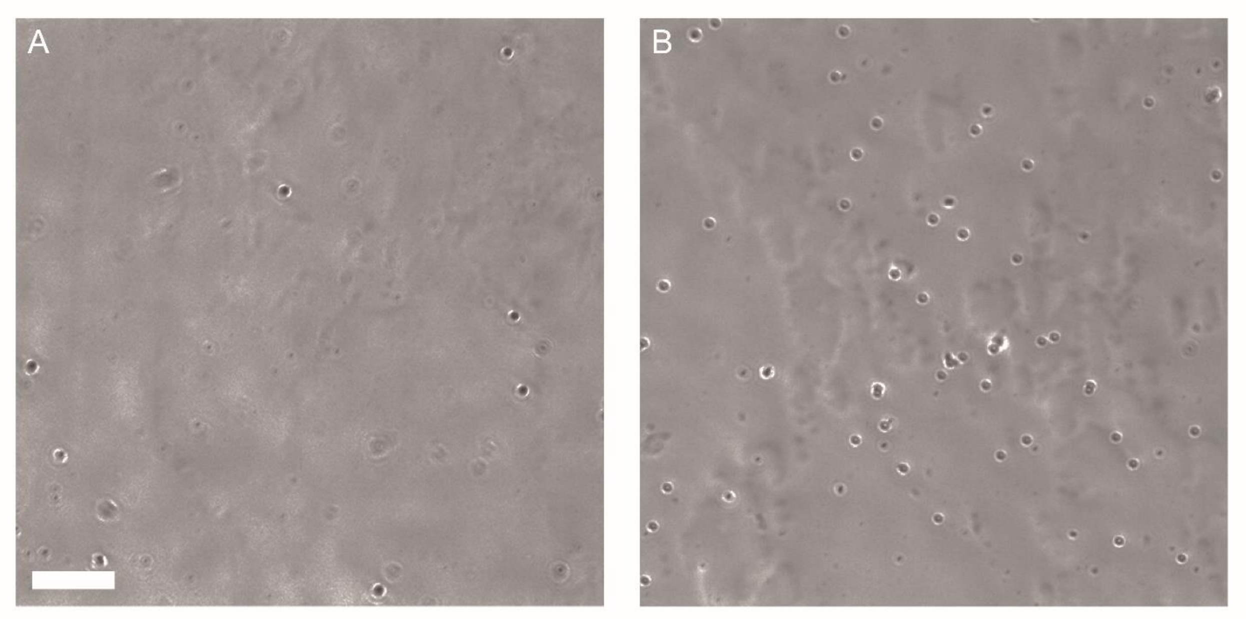

Supplementary Figure 4. Fibronectin conjugation of gradient ligand patterned DMNBAT-HA-methacrylate hydrogels. Fibronectin was passively adsorbed to latex beads prior to conjugation to the gel. Phase-contrast microscopy at a magnification of 20x was used to visualize bead density. Scale bar, 50 microns.

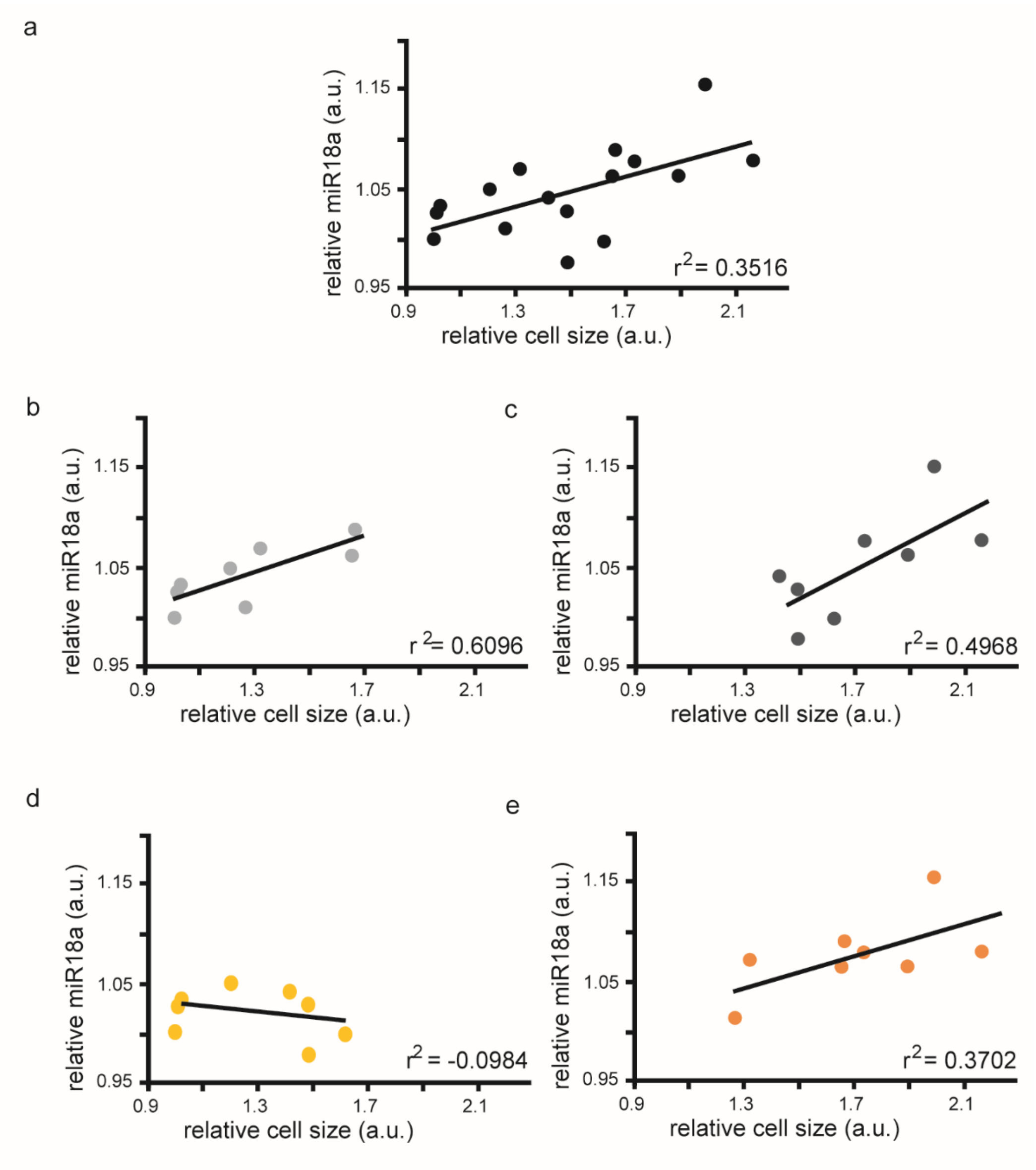
a
relative miR18a (a.u.)
1.15
1.05
0.95
0.9
1.3
1.7
2.1
relative cell size (a.u.)
r2= 0.3516
b
relative miR18a (a.u.)
relative cell size (a.u.)
r 2= 0.6096
c
relative miR18a (a.u.)
relative cell size (a.u.)
r2= 0.4968
d
relative miR18a (a.u.)
relative cell size (a.u.)
r2 = -0.0984
e
relative miR18a (a.u.)
relative cell size (a.u.)
r2= 0.3702

Supplementary Figure 5. Plotting the relative miR18a expression levels against the corresponding relative cell spread size for each stiffness-ligand combination reveals a positive correlation between miR18a levels and cell size (a). The data was then grouped into either low fibronectin (b) or high fibronectin (c) and the correlation between miR18a and cell size was plotted for conditions. For either high or low fibronectin densities, there is a strong correlation between miR18a expression levels and cell size. This analysis was repeated by grouping the data into either low or high substrate stiffness. Interestingly, mir18a levels are decoupled from cell size on soft substrates (d), but not on stiff substrates (e).

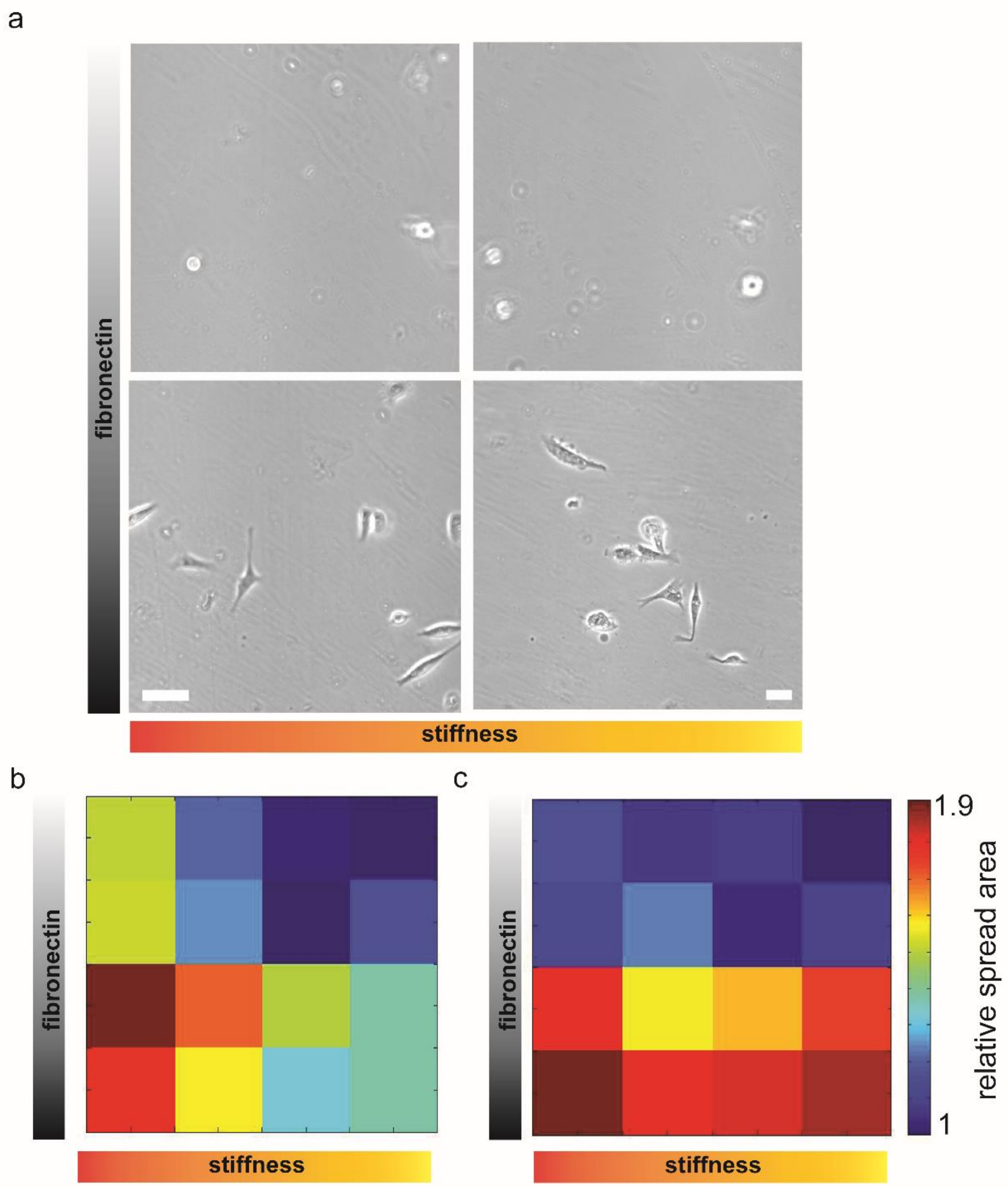

Supplementary Figure 6. Quantification of cell spread area in cells cultured with macrophage conditioned media. Phase contrast images reveal that cells fail to spread on low fibronectin conditions, regardless of substrate stiffness. Similarly, cells are highly spread when adhered to high fibronectin concentrations irrespective of substrate stiffness (a)Cell spread area was manually calculated at 16 difference positions on dual-patterned hydrogels in cells cultured in normal media (b) or media that was conditioned for 4 days by C8-B4 macrophages (c). N>20 for each stiffness-fibronectin density position. Scale bar, 50 microns.

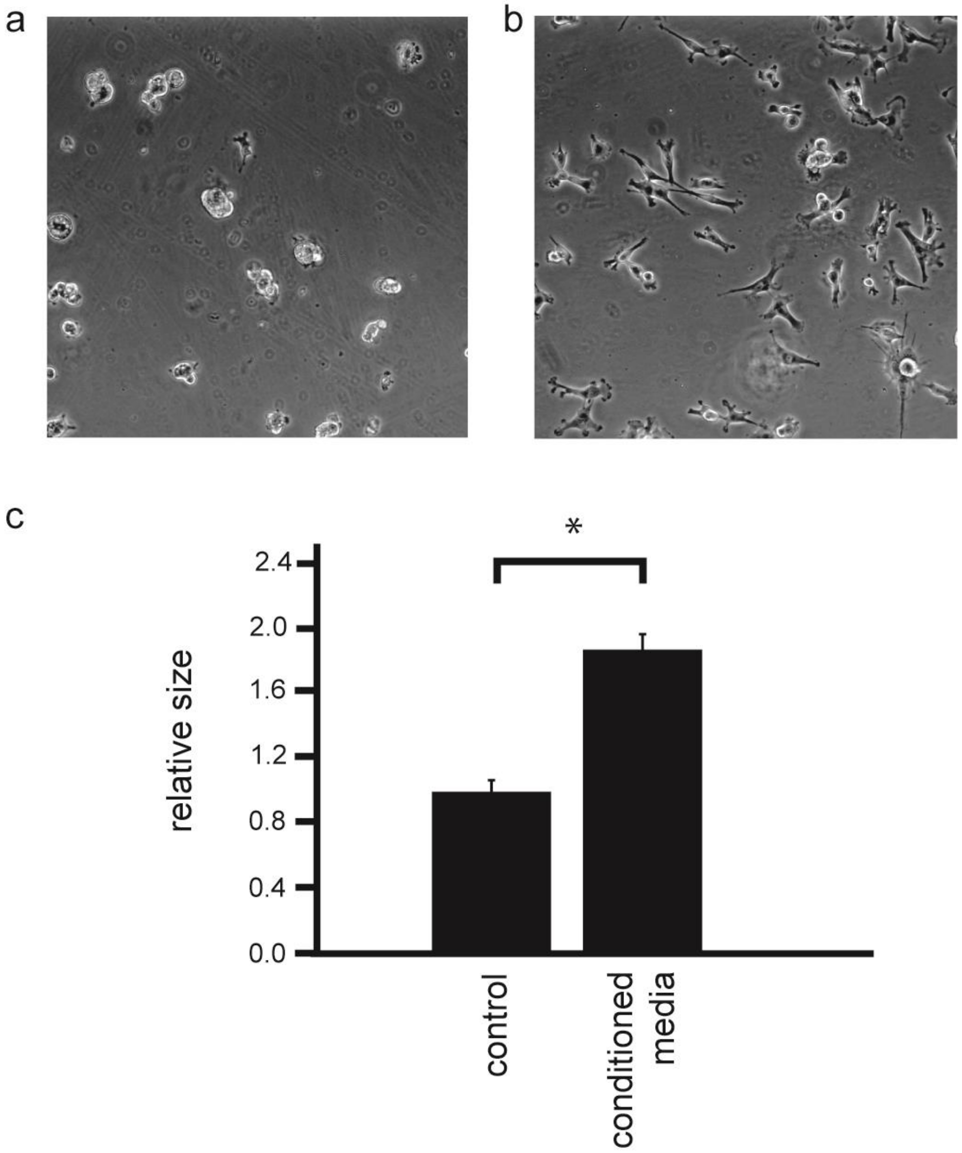

Supplementary Figure 7. Effects of macrophage conditioned media on spreading of U373-MG cells. Uniform gels were created with a high fibronectin concentration and low stiffness by exposing DMNBAT-HA-methacrylate gels to UV light through a uniformly 100% transparent photomask to create a high fibronectin concentration and omitting the second, light-based cross-linking step to create a uniformly soft hydrogel. These gels were then seeded with U373-MG cells. After cell adhesion to the gel, the cell culture medium was replaced with C8-B4 macrophage conditioned medium. After two days, phase contrast images were acquired for cells in normal U373-MG medium (a) or macrophage conditioned medium (b). In contrast to cells in normal medium, which were mostly rounded, cells cultured in macrophage-conditioned medium exhibited a spread, spindle-like morphology on this soft hydrogel. Quantification of cell area

revealed that cells in macrophage-conditioned medium displayed an approximately 2-fold increase in spread relative to cells in control medium. *=p<.0001. Errors bars are S.E.M.
